# Radiofrequency identification tag localization of nonpalpable breast lesions: a systematic review and meta-analysis

**DOI:** 10.1186/s41747-025-00657-z

**Published:** 2025-12-22

**Authors:** Gordon R. Daly, Mohammad Alabdulrahman, Gavin P. Dowling, Cian Hehir, Hayley Briody, Sami Almasri, Nuala A. Healy, Arnold D. K. Hill

**Affiliations:** 1 Department of Surgery, Royal College of Surgeons in Ireland, Dublin, Ireland; 2 Department of Surgery, Beaumont Hospital, Dublin, Ireland; 3 Department of Radiology, Royal College of Surgeons in Ireland, Dublin, Ireland; 4 Department of Radiology, Beaumont Hospital, Dublin, Ireland

**Keywords:** Breast neoplasms, Margins of excision, Radio waves, Reoperation, Radiology (interventional)

## Abstract

**Objective:**

Preoperative localization of nonpalpable breast lesions has traditionally been performed by wire-guided localization (WGL). Radiofrequency identification (RFID) tag localization provides a less invasive alternative. The aim of this systematic review and meta-analysis was to investigate outcomes of RFID in terms of clinical utility, efficacy, and safety, also in comparison with WGL.

**Materials and methods:**

Following PRISMA guidelines, studies reporting on outcomes post-RFID tag localization, and comparing outcomes post-RFID tag localization and WGL were included. Positive margins and re-excision rates were estimated using meta-analyses of proportions. Further meta-analyses compared positive margins and re-excision rates between RFID tag localization and WGL. Random effects models were used.

**Results:**

Nineteen studies involving 3,234 patients were included. Localization was performed for 497 benign and 2,741 malignant lesions. No study reported failure to retrieve an inserted RFID tag. Failed localization rates ranged from 0.0 to 60.7% across studies. After RFID tag localization, the pooled rate of positive margins was 12% (95% confidence interval (CI) 10–15%) and the pooled re-excision rate was 13% (95% CI 10–16%) in 14 and 16 studies, respectively; heterogeneity was high, *I*^*2*^ = 54.6% and 54.9, respectively. In three comparative studies, RFID tag localization was associated with significantly lower rates of positive margins than WGL, odds ratio (OR) 0.71 (95% CI 0.54–0.95), *p* = 0.021; however, no difference was observed in re-excision rates, OR 1.13 (95% CI 0.88–1.45), *p* = 0.346. Heterogeneity was low in both analyses, *I*^*2*^ = 0.0%. Moderate bias was reported in 16/19 studies, serious bias in 3/19.

**Conclusion:**

RFID tag localization provides an effective alternative to WGL.

**Relevance statement:**

This systematic review shows that RFID tag localization of nonpalpable breast lesions provides a less invasive, safe and effective alternative to WGL-guided localization for selected patients, considering its higher cost. Randomized trials are required to elucidate the benefit of RFID tag localization over WGL and other non-wire localization techniques.

**Key Points:**

- The pooled rates of positive margins and re-excision after RFID tag localization were 12% and 13%, respectively.
- RFID localization had significantly lower positive margin rates than wire-guided localization (WGL); however, no difference was observed in re-excision rate.
- RFID localization provides an effective alternative to WGL and may be of benefit in selected patients.
- Randomized trials are required to better elucidate the benefit of RFIS tag localization.

**Graphical Abstract:**

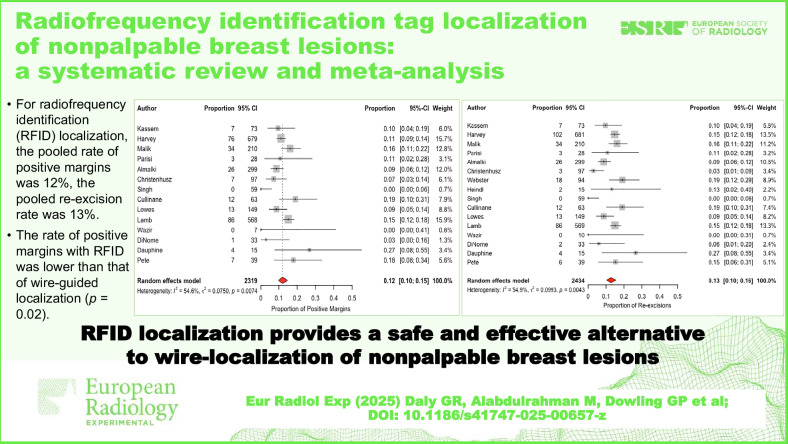

## Background

The detection of nonpalpable breast cancer has increased due to advancements in imaging technology and the implementation of population-based screening programs [1]. Consequently, a significant number of patients are now suitable for breast-conserving surgery (BCS) combined with radiotherapy, which has been shown to be as effective as mastectomy in terms of overall survival rates [2]. In BCS, precise intraoperative localization of the lesion is crucial to ensure clear surgical margins while preserving surrounding healthy tissue, thereby achieving optimal cosmetic and oncological results [3].

Traditionally, wire-guided localization (WGL) has been used for localizing breast lesions prior to local excision [4, 5]. However, given its numerous drawbacks, including scheduling inflexibility, wire displacement, patient discomfort, needle-stick injury risks, and potential cosmetic issues, its use is continually diminishing in clinical practice, with a paradigm shift toward the use of less invasive breast lesion localization devices [6–8].

A number of wireless localization techniques have been developed, each with distinct benefits and limitations. These include radioactive seed localization, magnetic and paramagnetic localization systems, and radar reflector-based systems [6, 9, 10].

Radiofrequency identification (RFID) tags represent another advanced option. These tags offer the advantage of individual tag differentiation through unique identification numbers, enhancing precision during surgery, in particular when multiple lesions should be localized [6]. They maintain signal integrity over time and allow for flexible scheduling. Despite these advantages, RFID systems share common drawbacks with some of the other wireless techniques, including high costs, generation of artifacts on magnetic resonance imaging (MRI), and limitations in repositioning after placement [6, 9, 11].

Despite its widespread application, RFIDs’ accuracy, associated positive margins and re-excision rates have been variably reported in the literature. In this systematic review and meta-analysis, we aimed to assess the clinical utility, efficacy, and safety of RFID tag localization and compare it to wire-guided localization of nonpalpable breast lesions, also in comparison with WGL.

## Materials and methods

This systematic review was performed in accordance with the Preferred Reporting Items for Systematic Reviews and Meta-Analyses (PRISMA) guidelines [12].

### Search strategy

An electronic search for relevant studies was conducted in August 2024 using Medline Ovid, CINAHL and Cochrane Library. Search terms included ‘radiofrequency identification,’ ‘tag’ and ‘breast neoplasm.’ Search terms were linked with Boolean Operators ‘AND’ and ‘OR.’ The search was limited to studies published in the previous 10 years. A detailed outline of the search strategy can be found in Supplementary Table S1.

### Eligibility criteria

This systematic review included studies that reported on outcomes after RFID tag insertion in both benign and malignant nonpalpable breast lesions. Inclusion criteria were: prospective and retrospective studies; studies reporting retrieval rates, localization failure or complications after RFID tag insertion; studies reporting positive margins or re-excision rates post localization; studies where RFID tag localization was compared to wire-guided localization or other localization technique. Exclusion criteria included: non-English language articles; case reports, and case series of < 10 patients, studies published > 10 years previously.

These parameters were chosen for inclusion as they comprise the main considerations influencing clinicians in selecting a localization technique [11, 13]. Case reports, small case series and older studies were excluded to increase the robustness and clinical applicability of this meta-analysis in 2025 [14].

### Study selection

**Fig. 1:**
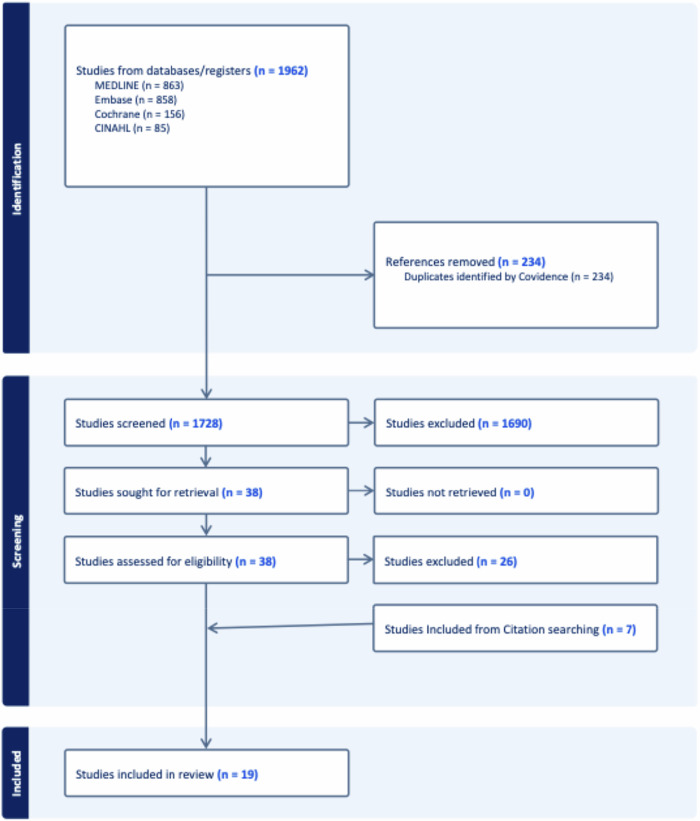
PRISMA flow diagram summarizing study inclusion

Two independent reviewers (G.R.D.) and (M.A.) performed the literature search. Duplicates were removed manually using Endnote. Titles and abstracts were screened using Covidence (https://www.covidence.org/), followed by a full-text review of studies that were deemed eligible. In instances of disagreement, a third reviewer (G.P.D.) adjudicated on the article’s inclusion. In two instances, articles used overlapping patient cohorts: Webster et al [14] and McGugin et al [15]; Lee et al [16] and DiNome et al [17]. Only one of each was included in each meta-analysis. A summary of study selection is provided in Fig. 1.

### Risk of bias

Risk of bias was assessed using the ROBINS-I tool [15].

### Statistical analysis

A meta-analysis of proportions was performed to analyze re-excision and positive margin rates across studies analyzing RFID tag localization only using a random effects model. Forest plots were generated using the meta package of “R” [16]. The significance of the overall effect was calculated using a Z-test. Heterogeneity was assessed using *I*^*2*^, and variance across studies was represented by τ^2^. *I*^*2*^ > 50% was considered as high heterogeneity [17]. Comparative meta-analysis of positive margins and re-excision rate post-RFID tag localization *versus* wire-guided localization was performed using a random effects model, with summary estimates made using the Mantel-Haenszel, on “Review Manager” version 5.4 (https://revman.cochrane.org). Point effects of estimates from dichotomous outcomes were described using a 95% confidence interval. A *p*-value of < 0.05 was considered significant for all analyses.

## Results

### Study and patient characteristics

A total of 19 studies involving 3,234 patients were included for qualitative assessment. Seven different countries were represented. Studies included 497 benign lesions and 2,741 malignant lesions (Table 1). There were 4 patients with > 1 lesion included. No study reported failure to retrieve an inserted RFID tag. Failed localization rates ranged from 0.0 to 60.7% across studies. Both positive margins and re-excision rates ranged from 0.0 to 26.7% across studies (Table 2).

**Table 1:** Summary of included studies

Fist author [reference]	Journal	Year	Country	Study design	Inclusion	Exclusion	Number of patients	RFID *vs* WGL	Age of patients (years)	Number of tags	Benign lesions	Malignant lesions
Kassem [30]	*European Journal of Breast Health*	2024	USA	Retrospective cohort	Patients undergoing BCS for nonpalpable BC	Patients with incomplete clinical data and those treated at other institutions were excluded	73	No	66.8*b* (30–91)*e*	73	0 (0%)	73 (100%)
Harvey [31]	*British Journal of Surgery*	2024	UK	Prospective cohort	Patients undergoing BCS for nonpalpable benign and malignant breast lesions in an 18-month study period	Surgeon having previously performed < 5 cases	1109	Yes	61.2*b* ± 10.2*c*	1109	109 (10.1%)	971 (89.9%)
Malik [32]	*The Surgeon*	2024	UK	Mixed prospective and retrospective cohort	Patients undergoing BCS for nonpalpable BC	NR	217	No	60*a* ± 14.11*e*	220	0 (0%)	220 (100%)
Parisi [19]	*Journal of Clinical Medicine*	2023	Italy	Retrospective cohort	Patients undergoing BCS for T1, nonpalpable BC	Multicentric extension of microcalcifications, benign lesions	28	No	58.3*a* ± 5.4*c*	28	0 (0%)	28 (100%)
Almalki [33]	*The Breast*	2023	UK	Prospective cohort	Patients with nonpalpable indeterminate lesions, DCIS or invasive BC	Patients receiving NAC	299	No	NR	312	6 (2%)	293 (98%)
Christenhusz [1]	*Breast Cancer Research and Treatment*	2023	Netherlands	Prospective cohort	Patients undergoing BCS for nonpalpable BC	Lesion depth > 7 cm, pregnancy or lactating status, and multicentric breast cancer, receiving NAC in some study sites	96	No	62*b* (54–69)*d*	100	0 (0%)	100 (100%)
Webster* [34]	*Journal of the American College of Surgeons*	2022	USA	Retrospective cohort	Patients who underwent BCS for nonpalpable benign and malignant breast lesions	Bilateral and multicentric lesions	147	Yes	59.5*a* (25–95)*e*	147	53 (36.1%)	94 (63.9%)
McGugin* [35]	*Breast Cancer Research and Treatment*	2019	USA	Retrospective cohort	Patients undergoing BCS with or without SLNB or excisional biopsy for nonpalpable breast lesions	Bilateral and multicentric lesions	147	No	59.5*a* ± 14.0*c*	147	53 (36.1%)	94 (63.9%)
Heindl [36]	*In Vivo*	2022	Germany	Prospective cohort	Patients undergoing BCS for nonpalpable breast lesions	NR	15	No	57.9*a*	16	2 (13.33%)	13 (86.67%)
Singh [37]	*Cureus*	2022	UK	Retrospective cohort	Patients undergoing BCS for nonpalpable BC	Benign lesions	59	No	NR	59	0 (0%)	59 (100%)
Cullinane [38]	*Surgical Innovation*	2021	Ireland	Prospective cohort	Patients undergoing BCS for nonpalpable indeterminate lesions, DCIS or invasive BC	NR	69	No	56*a* (50–65)*e*	69	6	63
Lowes [28]	*Clinical Radiology*	2020	UK	Prospective cohort	First 150 patients to undergo BCS for non-invasive BC	Benign lesions	150	No	62.1*a* (35–89)*e*	177	0 (0%)	177 (100%)
Lamb [29]	*American Journal of Roentgenology*	2020	USA	Retrospective cohort	All patients who underwent RFID localization of nonpalpable breast lesions	Nil	848	No	60*a* (23–86)*e*	1013	279 (32.9%)	568 (67%)
Wazir [8]	*In Vivo*	2020	UK	Prospective cohort	Patients undergoing BCS for nonpalpable BC	NR	10	No	52.9*a* (40–68)*e*	11	4 (36.4%)	7 (33.6%)
Malter [39]	*In Vivo*	2019	Germany	Prospective cohort	Patients undergoing excision of nonpalpable benign breast lesions	Malignant lesions	4	No	40.7*a* ± 10.3*c*	4	4 (100%)	0 (0%)
DiNome** [40]	*Breast Cancer Research and Treatment*	2019	USA	Prospective cohort	Patients undergoing BCS for nonpalpable BC	Patients who were pregnant, breast-feeding, multicentric, or Stage IV disease	50	No	59.2*a*	50	17 (34%)	33 (66%)
Lee** [41]	*Breast Cancer Research and Treatment*	2020	USA	Retrospective cohort	Patients who underwent BCS for nonpalpable BC	Benign lesions	33	Yes	61.3*a* ± 11.4 *c*	33	0 (0%)	33 (100%)
Dauphine [42]	*American Journal of Roentgenology*	2015	USA	Prospective non-randomized interventional study	Patients undergoing BCS for nonpalpable breast lesions	Depth of lesion was > 6 cm, palpable or defibrillator device was present	20	No	53*a* (38–64)*e*	20	5 (25%)	15 (75%)
Pete [20]	*BMC Cancer*	2025	France	Prospective non-randomized observational study	Patients undergoing BCS nonpalpable BC or high-risk lesions	Multiple breast lesions or multiple localizations, pregnancy	40	Yes	61*a* ± 12*c*	40	12 (31%)	27 (69%)

*a* Mean, *b* Median, *c* Standard deviation, *d* Interquartile range, *e* Range

*BCS* Breast-conserving surgery, *BC* Breast cancer, *SLNB* Sentinel lymph node biopsy, *NR* Not reported, *NAC* Neoadjuvant chemotherapy

* Separate studies of the same patient cohort

** Patient cohorts overlap

**Table 2:** Summary of outcomes after radiofrequency identification tag localization

First author [reference]	Failed localization	Failed retrieval	Complications	Positive margins	Re-excision	Tag insertion to operation time (days)
Kassem [26]	0 (0%)	0 (0%)	NR	7 (9.6%)	7 (9.6%)	NR
Harvey [27]	20 (2.1%)	NR	NR	76 (11.2%)	102 (15.0%)	NR
Malik [28]	10 (4.5%)	0 (0%)	NR	34 (16.2%)	34 (16.2%)	NR
Parisi [20]	17 (60.7%)	0 (0%)	NR	3 (10.7%)	3 (10.7%)	15 (all patients)
Almalki [29]	3 (1%)	0 (0%)	5 (1.7%)	26 (8.7%)	26 (8.7%)	21*b* (0–233)*e*
Christenhusz [1]	8 (8%)	0 (0%)	11 (11.5%)	7 (7.2%)	3 (3.1%)	7*b* (4–12)*d*
McGugin [14]	NR	0 (0%)	NR	NR	18 (19.1%)	1–22*e*
Heindl [30]	NR	NR	NR	NR	2 (13%)	0–7*e*
Singh [31]	0 (0%)	0 (0%)	14 (23.7%)	0 (0%)	0 (0%)	NR
Cullinane [32]	0 (0%)	0 (0%)	1 (1.4%)	12 (19%)	12 (19%)	NR
Lowes [34]	3 (1.7%)	0 (0%)	28 (26.7%)	13 (8.7%)	13 (8.7%)	7.8*a* (0–71)*e*
Lamb [25]	16 (2.8%)	0 (0%)	0 (0%)	86 (15.1%)	86 (15.1%)	3*b* (1–188)*e*
Wazir [8]	0 (0%)	0 (0%)	NR	0 (0%)	0 (0%)	NR
Malter [33]	0 (0%)	0 (0%)	0 (0%)	NA	NA	NR
DiNome [16]	0 (0%)	0 (0%)	NR	1 (3%)	2 (6.1%)	1.4*a* ± 2.8*c*
Dauphine [34]	0 (0%)	0 (0%)	0 (0%)	4 (26.7%)	4 (26.7%)	NR
Pete [23]	1 (2.5%)	0 (0%)	0 (0%)	7 (17.9%)	6 (15.4%)	NR

Numbers represent values reported in individual studies

*a* Mean, *b* Median, *c* Standard deviation, *d* Interquartile range, *e* Range

*NR* Not reported, *NA* Not applicable

### Meta-analysis of positive margins and re-excision rates post-RFID tag localization

**Fig. 2:**
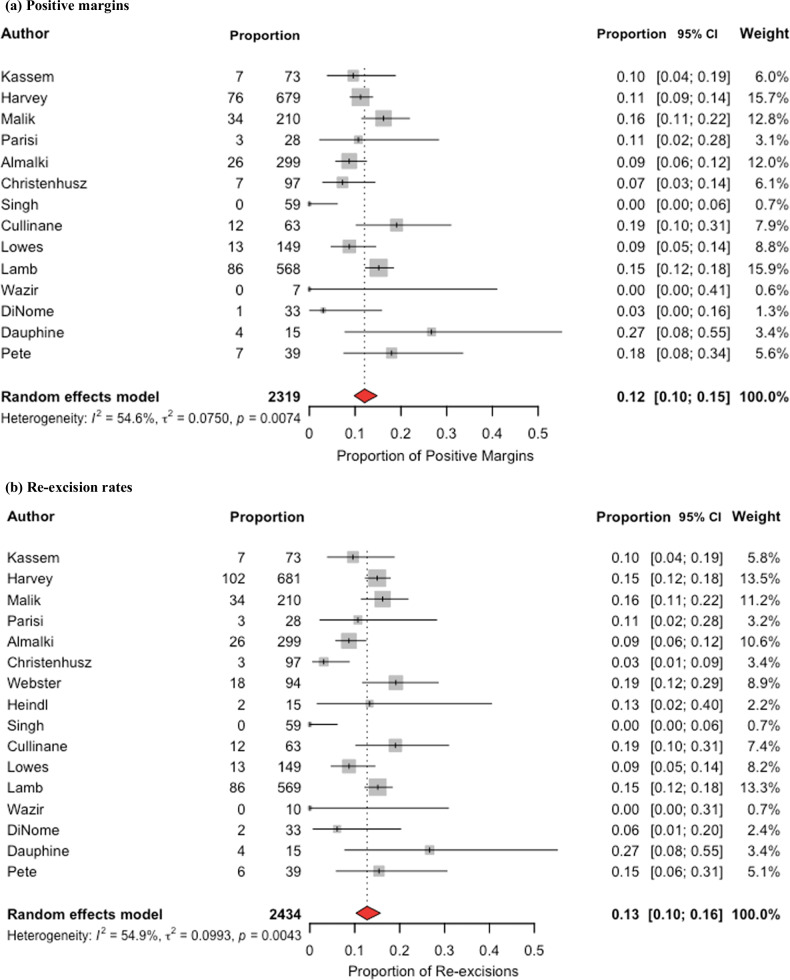
Meta-analysis of proportions of positive margins and re-excision rates after radiofrequency identification tag localization. **a** Positive margins. **b** Re-excision rates

Meta-analysis of proportions for positive margins post-RFID-tag included 14 studies. The pooled rate of positive margins was 12% (95% CI 10–15%), *p* = 0.007. Heterogeneity was high, *I*^*2*^ = 54.6% (Fig. 2a).

Meta-analysis of proportions for re-excision rates post-RFID tag localization included 16 studies. The pooled re-excision rate was 13% (95% CI 10–16%), *p* = 0.004 across studies. Heterogeneity was high, *I*^*2*^ = 54.9% (Fig. 2b). The significant *p-*value suggests that the pooled estimate results were highly unlikely due to chance. Whereas, in the comparative meta-analysis that follows, the *p-*value represents the likelihood of the odds ratio or difference between the groups being due to chance.

### Meta-analysis of positive margins and re-excision rates post-RFID tag *versus* wire-guided localization

**Fig. 3:**
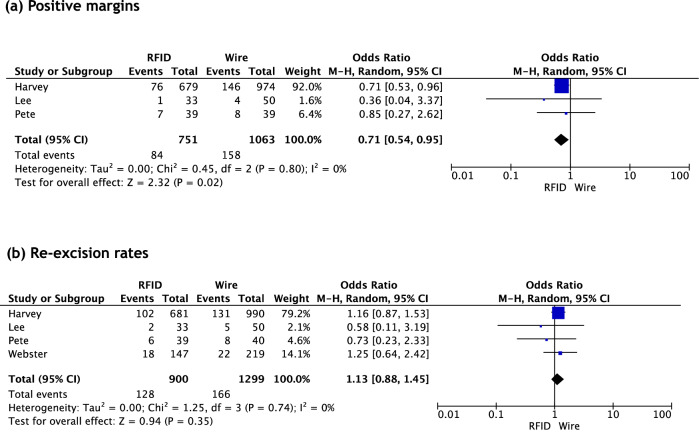
Comparative meta-analysis of positive margins and re-excision rates after radiofrequency identification tag localization (RFID) vs wire-guided localization. **a** Positive margins. **b** Re-excision rates

Pooling the data provided by three studies, RFID tag localization was associated with statistically significantly lower levels of positive margins than wire-guided localization, with an OR of 0.71 (95% CI 0.54–0.95), *p* = 0.021 (Fig. 3a). However, no significant difference was found in re-excision rates between techniques, with an OR 1.13 (95% CI 0.88–1.45) *p* = 0.346 (Fig. 3b). Heterogeneity was low in both analyses, *I*^*2*^ = 0.0%.

### Risk of bias

Moderate bias was reported in 16 of 19 studies, while serious bias was identified in 3 of 19. Risk of bias was predominantly observed in Domain 1 (bias due to confounding) and Domain 6 (bias in measurement of outcomes). Summary in Supplementary Fig. S1.

## Discussion

This is the largest systematic review and meta-analysis of outcomes after RFID tag localization and includes the first analysis of RFID *versus* WGL in the literature to date. The key findings were the following: (1) both positive margin and re-excision rates were low across all studies following RFID localization (12% and 13%, respectively); and (2) while RFID tag localization was associated with significantly lower positive margin rates compared to WGL (OR 1.71), this did not translate to a reduction in number of surgical re-excisions (OR 1.13). As the included data are observational, our confidence in the estimated effect is limited. Definitive assessment awaits evidence from randomized controlled trials RCTs.

A recent network meta-analysis of RCTs comparing WGL to other localization techniques found that re-excision rates ranged from 4.8 to 18.9%, and positive margins from 5.4 to 28.2% for all techniques [13]. Unfortunately, no RCT has been performed involving RFID tag localization to date, limiting clinicians’ ability to accurately quantify its true value and identify the specific patients who will benefit from its use in the era of personalized medicine. However, the pooled re-excision rate of 13% and positive margins rate of 12% in this meta-analysis of non-randomized studies suggest that RFID tag localization has outcomes similar to those of other techniques. Positive margins is a key comparative metric for localization techniques as it provides a pathologically confirmed assessment of their accuracy in correctly localizing cancerous tissue. Complete cancer excision improves oncological outcomes by reducing tumor burden and reducing the requirement for re-operation [18].

Additionally, low rates of complications were reported across RFID studies. Other clear benefits include that RFID does not involve exposing patients to ionizing radiation and ease of use, meaning it requires only minimal training. While patient’s, surgeon’s, and radiologist’s satisfaction and cosmetic results were not widely reported across studies, it was generally positive [18–20]. This is unsurprising given the evident ergonomic benefit of the RFID tag compared to guidewire. Wazir et al [8] reported a mean visual analog scale score of 9.9/10 across just 7 patients post-RFID tag localization. Compared to the use of the RFID localization system only, Parisi et al [19] found that ultrasound confirmation of RFID placement immediately after insertion improved patient and clinician Likert scores from 8.1/10 and 7.8/10 to 9.2/10 and 7/10, respectively. In a non-randomized prospective study [20], at 1-month follow-up, in 40 patients who underwent RFID localization compared to 40 patients post WGL, visual analog scale scores were 10/10 *versus* 9.8/10 respectively. While statistically significant, this assessment did not elicit a clinically significant difference between techniques.

Another benefit of RFID tag localization is that it may be performed weeks prior to surgery, in contrast to WGL, which requires the availability of a radiologist on the day of surgery. This is not a universal benefit across other minimally invasive techniques, such as intraoperative ultrasound, which requires a radiologist to be present during the case or additional training for the surgeon [21, 22]. Whether this scheduling benefit translates into a cost benefit requires further economic evaluation, particularly as the cost of the RFID tag system itself is considerably greater than that of the wire, with a reported cost of $20 for the wire and $550 for the RFID system [23], *i.e*., over 27-fold increase. Selected patient cohorts may receive the most benefit from RFID localization. For example, each RFID tag has a unique identifier, allowing identification of multiple lesions in those with multifocal disease. Additionally, the increased scheduling flexibility afforded by RFID localization aids patients traveling long distances to the hospital.

A number of alternative wireless localization techniques have been developed, each with distinct benefits and limitations. In terms of margins and re-excision, our findings suggest RFID’s performance is in line with other techniques [13, 24]. Similar to RFID localization, radioactive seed localization offers scheduling flexibility yet is restricted by radiation safety concerns, regulatory restrictions in some regions, and the challenges of handling radioactive materials [6, 9]. Magnetic and paramagnetic localization systems eliminate radiation exposure risks and also offer scheduling flexibility [6, 9]. These systems are advantageous for their reliable signal detectability over extended periods, suitable for patients undergoing neoadjuvant therapy [6]. However, they pose challenges, including artifacts on MRI, interference with metal surgical tools, and potential issues with patients having pacemakers or defibrillators [6, 10]. Radar reflector-based localization systems provide similar benefits to radioactive seed localization and magnetic localizers, including reliable long-term signal detection [6, 25]. An advantage of them is minimal associated MRI artifacts, so that MRI can be performed following insertion of this device. Intraoperative ultrasound has shown very low positive margin and re-excision rates in RCTs (often < 5%), making it a highly effective method when lesions are sonographically visible. RFID cannot compete with intraoperative ultrasound in those cases, but can be used for lesions not visualized at (intraoperative) ultrasound [26, 27].

Despite the positive aspects of RFID tag localization, several limitations are evident. The depth of the tumor is a key consideration as RFID localization is not suitable for deep (> 60 mm) or superficial (< 5 mm) lesions [20]. A risk of tag migration requiring a second tag or a wire insertion has been reported in a number of studies [28, 29]. Lowes et al [28] reported the RFID device misfiring and not reaching or overshooting the intended lesion, underscoring the importance of an individualized patient-specific approach when selecting a localization technique. Furthermore, the RFID tag may cause artifacts on MRI, necessitating tag placement after completion of neoadjuvant therapy and prior to surgery if used in the neoadjuvant setting [11].

Regarding the results of this systematic review, we should consider the substantial heterogeneity in outcomes across studies, likely due to differences in case mix and definitions. Consequently, the pooled 12–13% rates should be interpreted with caution: individual institutional outcomes may vary. Most included studies had a retrospective design, and those that were performed prospectively were not randomized. Therefore, the selection of patients and cases to undergo RFID tag localization likely differed across studies, leading to high levels of heterogeneity. Other likely factors contributing to heterogeneity were the differences in sample sizes and discrepancies in the experience of clinicians in using the technique. Additionally, the definition of positive margins varied between studies and was not defined in many cases, leading to outcome variation and limiting comparability.

In addition, failed localization was not reported, reported differently using different definitions, or poorly defined across studies. As subsequent treatment decisions were made by the operating surgeon, they may not have matched treatment guidelines in many cases. This likely explains the discordance between positive margins and re-excision rates, particularly where surgeons may be less stringent about margin adequacy for ductal carcinoma *in situ*. Data on the length of operation, time from RFID tag insertion to operation, specimen size, and patient’s and clinician’s satisfaction were not widely reported across studies. Furthermore, one included study reported an unusually high localization failure rate (~ 60%), which skews the range; this might reflect early technical issues not representative of current practice. Finally, most studies were performed in high-volume academic institutions with access to specialized breast radiologists, surgeons and suitable cases. This may limit the real-world applicability of the data, where factors like cost and training may influence results.

Finally, we note that we compared RFID to WGL as is common in most studies of minimally invasive localization techniques. However, given the declining use of WGL, future research efforts must focus on comparing alternative localization techniques that have widely replaced WGL. For example, MELODY (NCT05559411) is an ongoing prospective multicenter clinical study currently evaluating RFID tag localization, among several other non-wire localization techniques, across 30 countries [6]. With a target accrual of 7,416 patients, this study will provide significant insight into the benefits of various localization alternatives to WGL [6]. Beyond oncologic safety, patient and clinician satisfaction are recorded, and an economic evaluation is performed [6].

In conclusion, this systematic review of 19 studies including 3,234 patients, showed that RFID tag localization is a safe, feasible and effective method for preoperative localization of nonpalpable breast lesions, with a pooled rate of positive margins of 12% and a pooled re-excision rate of 13% It offers a minimally invasive, radiation-free alternative to WGL that can improve surgical scheduling. RFID may be particularly beneficial for patients requiring localization of multiple lesions or those who need localization performed well ahead of surgery for logistical reasons, as well as in centers aiming to eliminate the challenges associated with wires. Prospective RCTs comparing RFID localization to other modern alternatives are of growing importance as WGL use declines.

## Supplementary information

**Additional file 1: Table S1.** Detailed summary of search strategy. **Fig. S1.** ROBINS-I risk of bias assessment.

## Abbreviations

BCS: Breast-conserving surgery
CI: Confidence interval
MRI: Magnetic resonance imaging
OR: Odds ratio
RCT: Randomized controlled trial
RFID: Radiofrequency identification
WGL: Wire-guided localization
