# Supplementary material for: Radiofrequency identification tag localization of nonpalpable breast lesions: a systematic review and meta-analysis
Source: Eur Radiol Exp. 2025 Dec 22;9:122. doi: 10.1186/s41747-025-00657-z (PMC12722611; doi:10.1186/s41747-025-00657-z)
Supplement: Supplementary file 1 — Additional file 1: Table S1. Detailed summary of search strategy. Fig. S1. ROBINS-I risk of bias assessment. [file 41747_2025_657_MOESM1_ESM.pdf]

Radiofrequency identification tag localization of nonpalpable breast lesions: a systematic review and meta-analysis

ELECTRONIC SUPPLEMENTARY MATERIAL

Supplemental Table S1. Detailed summary of search strategy.

	Ovid MEDLINE	*Results*
1	("radio frequency identif*" or "radio-frequency identif*" or ((tag adj2 locali?ation) or (RFID adj2 "tag system*")) or ("radioguided occult lesion locali?ation" or "ROLL" or "Radioguided surger*" or "Radio guided occult lesion locali?ation" or "ROLL" or "Radio guided surger*") or ("Radiofrequenc*" or "radar guided locali?ation*") or (tag* adj2 device*)).tw. or exp Radio Frequency Identification Device/ or (Magseed or "Savi Scout" or "Hologic LOCalizer").tw.	59,502
2	(breast adj2 (neoplasm* or cancer)).tw. or exp Breast Neoplasms/	460,237
3	1 and 2	***863***
4	limit 3 to english language	806
5	limit 4 to yr="2014 -Current"	***442***
	CINAHL	
1	TI ("radio frequency identif*" or "radio-frequency identif*" OR (tag N2 (localization or localisation)) OR (RFID N2 "tag system*") OR (MH "Radio Frequency Identification") OR "radioguided occult lesion localization" or "ROLL" or "Radioguided surger*" or "Radio guided occult lesion localization" or "ROLL" or "Radio guided surger*" or "Radiofrequenc*" or "radar guided localization*" or (tag* N2 device*) or "radioguided occult lesion localisation" or "Radio guided occult lesion localisation" or "radar guided localisation*") OR AB ("radio frequency identif*" or "radio-frequency identif*" OR (tag N2 (localization or localisation)) OR (RFID N2 "tag system*") OR (MH "Radio Frequency Identification") OR "radioguided occult lesion localization" or "ROLL" or "Radioguided surger*" or "Radio guided occult lesion localization" or "ROLL" or "Radio guided surger*" or "Radiofrequenc*" or "radar guided localization*" or (tag* N2 device*) or "radioguided occult lesion localisation" or "Radio guided occult lesion localisation" or "radar guided localisation*")	16,801
2	TI (Magseed or "Savi Scout" or "Hologic LOCalizer") OR AB (Magseed or "Savi Scout" or "Hologic LOCalizer")	33
3	S1 OR S2	16,832
4	(TI (breast N2 (neoplasm* OR cancer)) OR AB (breast N2 (neoplasm* OR cancer))) OR (MH "Breast Neoplasms+")	123,471
5	S3 AND S4	***211***
6	Limiters - Exclude MEDLINE records	***85***
7	Limiters - Publication Date: 20140101-20241231	77
8	Limiters - Publication Date: English Language	***77***
	Cochrane	

1	("radio frequency" NEXT/1 identif*) OR ("radio-frequency" NEXT/1 identif*)	28
2	(tag NEAR/2 locali?ation) OR (RFID NEAR/2 (tag NEXT/1 system*))	0
3	radioguided occult lesion localization or "ROLL" or (Radioguided NEXT/1 surger*) or "Radio guided occult lesion localization" or "ROLL" or ("Radio guided" NEXT/1 surger*) or Radiofrequenc* or ("radar guided" NEXT/1 localization*) or (tag* N2 device*) or "radioguided occult lesion localisation" or "Radio guided occult lesion localisation" or ("radar guided" NEXT/1 localisation*)	8,380
4	Magseed or "Savi Scout" or "Hologic LOCalizer"	11
5	MeSH descriptor: [Radio Frequency Identification Device] explode all trees	4
6	#1 OR #2 OR #3 OR #4 OR #5	8,401
7	breast NEAR/2 (cancer OR neoplasm*)	46,422
8	MeSH descriptor: [Breast Neoplasms] explode all trees	20,230
9	#7 OR #8	46,452
10	#6 AND #9	***157***
	Cochrane Reviews	10
	Custom Range: 01/01/2014 to 06/06/2024	8
	Trials	140
	Custom Range: 2014 to 2024	97
	Filter: Language: English	95
	Cochrane Protocols	6
	Custom Range: 2014 to 2024	4
	EMBASE	
1	radio frequency identif*':ti,ab OR 'radio-frequency identif*':ti,ab OR ((tag NEAR/2 locali?ation):ti,ab) OR ((rfid NEAR/2 'tag system*'):ti,ab) OR 'radio frequency identification device'/exp OR 'radioguided occult lesion locali?ation':ti,ab OR 'radioguided surger*':ti,ab OR 'radio guided occult lesion locali?ation':ti,ab OR 'roll':ti,ab OR 'radio guided surger*':ti,ab OR 'radiofrequenc*':ti,ab OR 'radar guided locali?ation*':ti,ab OR ((tag* NEAR/2 device*):ti,ab) OR magseed:ti,ab OR 'savi scout':ti,ab OR 'hologic localizer':ti,ab	90,335
2	((breast NEAR/2 (neoplasm* OR cancer)):ti,ab) OR 'breast cancer'/exp	713,546
3	#1 AND #2	***1,684***
4	#3 NOT [medline]/lim	858
5	#4 NOT AND [english]/lim	825
6	#5 AND [2014-2024]/py	***593***

Supplemental Figure S1. ROBINS-I risk of bias assessment.

a)

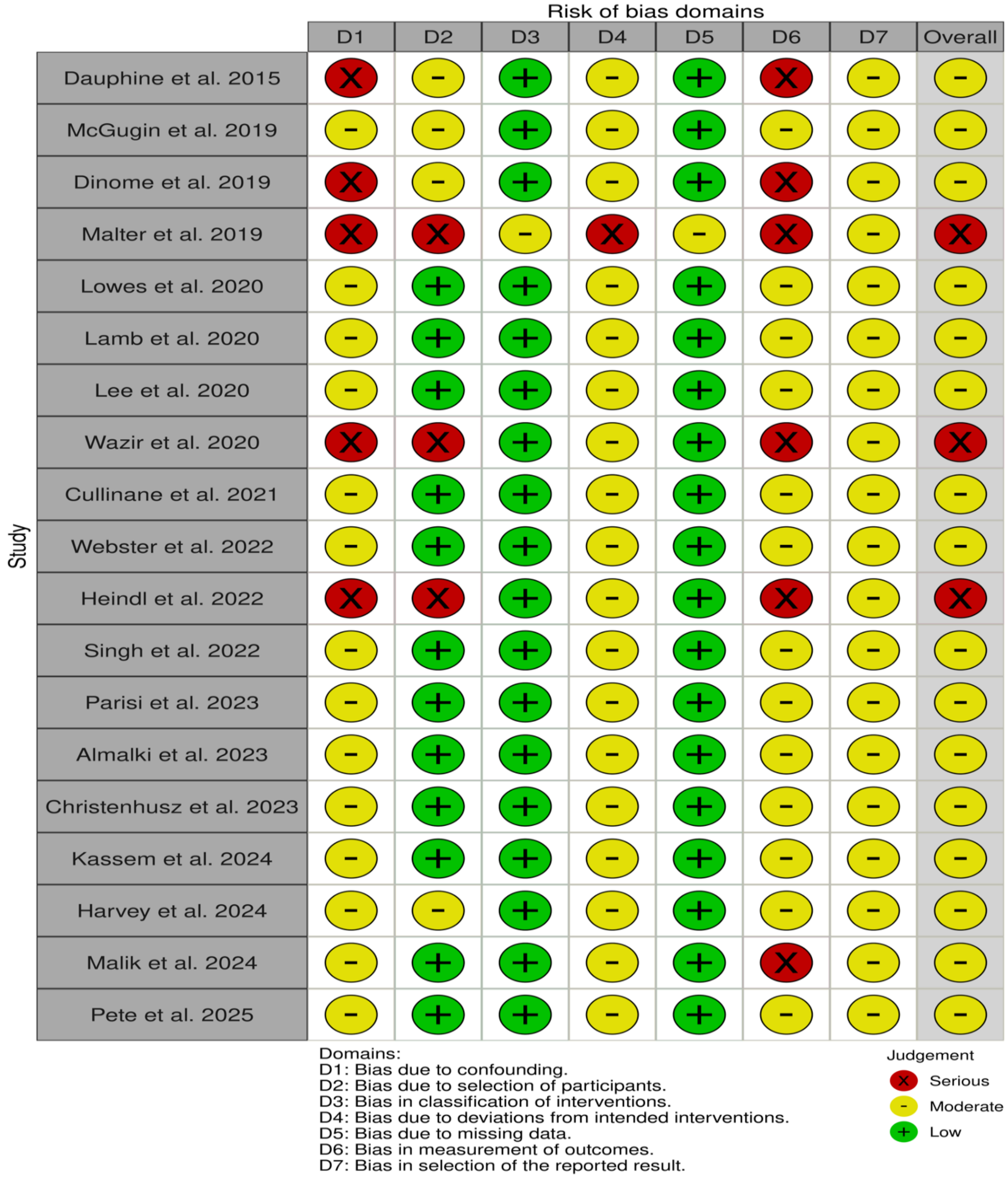

b)

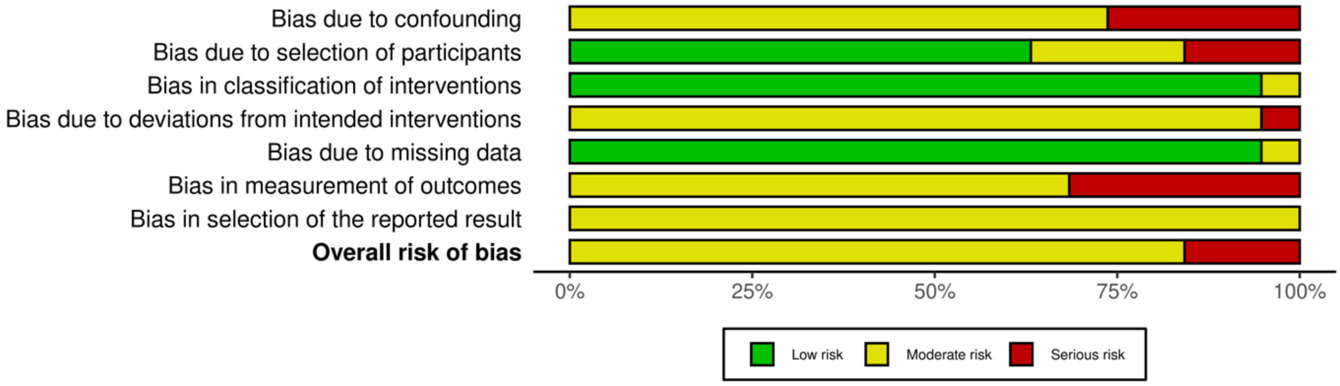
Bias due to confounding
Bias due to selection of participants
Bias in classification of interventions
Bias due to deviations from intended interventions
Bias due to missing data
Bias in measurement of outcomes
Bias in selection of the reported result
Overall risk of bias
0%
25%
50%
75%
100%
Low risk
Moderate risk
Serious risk
